# Spinal cysticercosis: a rare cause of myelopathy

**DOI:** 10.1186/s12883-022-02589-2

**Published:** 2022-02-22

**Authors:** Chenlong Yang, Tie Liu, Jian Wu, Jingcheng Xie, Tao Yu, Wenqing Jia, Jun Yang, Yulun Xu

**Affiliations:** 1 Department of Neurosurgery, Peking University Third Hospital, Haidian District, Beijing, China; 2 North America Medical Education Foundation, Union, CA USA; 3 Department of Neurosurgery, China National Clinical Research Center for Neurological Diseases, Beijing Tiantan Hospital, Capital Medical University, Beijing, China; 4 Department of Ophthalmology, Stanford University School of Medicine, Palo Alto, CA USA

**Keywords:** Cysticercosis, Parasitic infection, Myelopathy, Neurocysticercosis, *Taenia solium*

## Abstract

**Background:**

Neurocysticercosis is a neuroinfectious disease caused by the larval stage of the tapeworm *Taenia solium*. Isolated spinal cysticercosis is rare, with limited cases having been reported in the literature. This entity poses great diagnostic and therapeutic challenges.

**Methods:**

This retrospective study included seven patients pathologically diagnosed with spinal cysticercosis. The clinical manifestations, radiological features on magnetic resonance imaging (MRI), treatment, and outcomes were analyzed.

**Results:**

This case series consisted of four male and three female patients, with an average age of 34.9 ± 10.9 years. Clinically, six patients manifested with localization-related myelopathy. There were four solid lesions, one cystic-solid lesion, and three cystic lesions. The solid and cystic-solid lesions showed characteristic MRI features: 1) within the lesion, there was a mural nodule with isointensity on T1WI and iso- to hyperintensity on T2WI; 2) the signals at the periphery of the mural nodule were variable, ranging from hypointense to hyperintense on T2WI; and 3) ring-like or cyst wall enhancement could be present, and dot-like enhancement could be noted in the mural nodule. Complete resection of the responsible lesion was achieved in all patients, and oral albendazole was administered in a patient with one more suspected homologous lesion. After a mean follow-up period of 56.7 ± 35.1 months, the patient’s symptoms mostly regressed.

**Conclusion:**

Spinal cysticercosis is an extremely rare cause of myelopathy. Characteristic MRI features can facilitate preoperative diagnosis. Clinicians should be aware of this entity, and it should be included in the differential diagnosis of myelopathy.

## Background

Cysticercosis caused by the larval stage of the tapeworm *Taenia solium* is a common parasitic infection of the central nervous system in humans [1]. According to historical data, the first case of neurocysticercosis can be traced back to 1558, which was described by Rumler during the autopsy of a patient with epilepsy, and Malpighi described the scolex of the *Taenia solium* inside the cerebral vesicles in 1697 [2]. During the nineteenth century, morphologic similarities between the head of the adult *Taenia solium* and the scolex of cysticercus were recognized [3]. In 1855, Küchenmeister first demonstrated that human intestinal taeniasis was caused by the ingestion of cysticercus from pork [4]. In 1933, Yoshino clarified the life cycle of this parasite by infecting himself with *Taenia solium* cysticerci [5, 6]. Currently, this disease is endemic in Latin America (including Mexico, Peru, and Brazil), sub-Saharan Africa, and some developing countries in Asia (such as China, Korea, India, and Indonesia), and it leads to more than 50,000 deaths annually in these areas [7]. Although cysticercosis is rare in North America and most European countries, its incidence in these developed countries has increased in recent years due to imported and immigrated cases from endemic areas [8]. Spinal cysticercosis represents a small minority of neurocysticercosis, and the frequency of spinal involvement in patients with neurocysticercosis was reported to vary from 0.25 to 5.8% [9]. Spinal cysticercosis can be classified anatomically into three types: extradural, intradural-extramedullary, and intramedullary. In previous literature, limited cases of spinal intramedullary cysticercosis have been reported, most of which have a cysticercosis history or concomitant brain cysticercosis [10]. Due to its extreme rarity, this entity poses great diagnostic and therapeutic challenges, and it is easily misdiagnosed as intramedullary tumors. In this study, we summarized the clinical and radiological profiles of a case series of isolated spinal cysticercosis.

## Materials and methods

### Patients

This retrospective study included seven patients pathologically diagnosed with primary spinal cysticercosis at the Peking University Third Hospital and Beijing Tiantian Hospital between 2010 and 2020. The clinical and radiological profiles were collected and analyzed.

### Radiological evaluation

Magnetic resonance imaging (MRI) profiles were available for all patients. The location, size, appearance, and signal characteristics of cysticercotic lesions on T1-weighted imaging (T1WI), T2-weighted imaging (T2WI), and contrast-enhanced imaging were analyzed.

### Treatment

Surgical resection of the lesion was performed in all patients via a posterior midline approach with the assistance of neuroelectrophysiological monitoring. The surgical records documenting the intraoperative findings were analyzed. Postoperatively, one patient was treated with oral albendazole.

### Follow-up

Follow-up data for all patients were obtained during individual office visits or telephone interviews, with a mean follow-up time of 56.7 ± 35.1 months (range 10–91 months). The clinical outcomes were evaluated, focusing on the neurological functions and symptoms of the patients compared with their preoperative status.

## Results

### Clinical characteristics

This case series consisted of four male and three female patients, with an average age of 34.9 ± 10.9 years (range, 23–50 years). The onset symptoms included local pain (4/7), sensorimotor disturbance (6/7), and sphincter dysfunction (1/7). The duration of symptoms preceding the surgery ranged from 2 months to 3 years (mean, 14.4 ± 15.1 months). All the cases were sporadic, and the patients denied any remarkable unhygienic diet or affected relatives. The clinical data are presented in Table 1.

**Table 1:** Clinical characteristics of patients with spinal cysticercosis

No.	Age (years)	Sex	Symptoms	Duration (month)	Location	Suspected diagnosis	Surgical treatment	Pharmacotherapy	Follow-up period (month)	Outcome
1	23	M	Burning pain in the bilateral thighs; numbness and weakness in lower extremities	12	T11-T12 (IM)	Cavernous malformation	Complete resection	–	91	Mild sensory impairment
2	24	M	Back pain; weakness of the left leg	36	T5 (IM)	Ependymoma	Complete resection	–	84	Normal
3	47	M	Numbness and weakness in lower extremities; dysuria and constipation	2	L1 (IM)	Enterogenous cyst	Complete resection	Albendazole (15 mg/kg) for 1 month	82	Normal
					T7-T8 (IM)	Infection of the spinal cord	–			
4	27	F	Back pain; weakness in lower extremities	6	L1-S1 (IM)	Arachnoid cyst	Complete resection	–	79	Normal
5	38	M	Numbness and weakness in lower extremities	6	T8 (IM)	Cavernous malformation	Complete resection	–	36	Normal
6	35	F	Numbness and weakness in lower extremities	3	T7 (IM)	Cavernous malformation	Complete resection	–	15	Mild sensory impairment
7	50	F	Pain in the back and lower extremities	36	T10-L1 (EM)	Cysticercosis	Complete resection	–	10	Normal

*M* male, *F* female, *IM* intramedullary, *EM* extramedullary

### Radiological features

A total of eight cysticercosis lesions were identified, involving the thoracic spinal cord in six cases, the lumbar region in three cases, and the sacral region in one case. The mean dimension of the lesions was 48.8 ± 56.5 mm (range, 13–143 mm). There were four solid lesions, one cystic-solid lesion, and three cystic lesions. The solid and cystic-solid lesions showed characteristic MRI features: 1) within the lesion, there was a mural nodule with isointensity on T1WI and iso- to hyperintensity on T2WI; 2) the signals at the periphery of the mural nodule were variable, ranging from hypointense to hyperintense on T2WI; 3) ring-like enhancement was seen in Case 1, cyst wall enhancement was demonstrated in Case 7, and dot-like enhancement was noted in the mural nodule in Case 2. The MRI features of the cystic lesion were nonspecific, showing hypointensity on T1WI and homogeneous hyperintensity on T2WI. The preoperative suspected diagnosis included cavernous malformation (3/7), ependymoma (1/7), enterogenous cyst (1/7), and arachnoid cyst (1/7). The radiological characteristics of spinal cysticercosis are summarized in Table 2.

**Table 2:** Radiological characteristics of spinal cysticercosis

Case no.	Diameter (mm)	Solid-cystic appearance	Spinal T1-weighted MRI	Spinal T2-weighted MRI	Spinal Gd-DTPA contrast-enhanced MRI	Brain MRI	Brain CT
1	17	Solid	Mural nodule: isointense Periphery: hypointense Surrounding parenchyma: isointense	Mural nodule: hyperintense Periphery: hypointense Surrounding parenchyma: hyperintense	Ring-like enhancement in the surrounding parenchyma	Normal	N.A.
2	13	Cystic-solid	Mural nodule: isointense Periphery: hypointense Surrounding parenchyma: normal	Mural nodule: iso- to hyperintense Periphery: hyperintense Surrounding parenchyma: normal	Dot-like enhancement in the mural nodule	Normal	Normal
3	L1: 35	Cystic	Mural nodule: invisible Cystic lesion: hypointense Surrounding parenchyma: normal	Mural nodule: invisible Cyst: hyperintense Surrounding parenchyma: normal	No enhancement	Normal	Normal
	T7-T8: 18	Solid	Mural nodule: invisible Solid lesion: isointense Surrounding parenchyma: normal	Mural nodule: invisible Solid lesion: hyperintense Surrounding parenchyma: normal	No enhancement	Normal	
4	143	Cystic	Mural nodule: invisible Cystic lesion: hypointense Surrounding parenchyma: normal	Mural nodule: invisible Cyst: hyperintense Surrounding parenchyma: normal	No enhancement	Normal	Normal
5	15	Solid	Mural nodule: isointense Periphery: hypointense Surrounding parenchyma: normal	Mural nodule: iso- to hyperintense Periphery: hyperintense Surrounding parenchyma: normal	No enhancement	Normal	N.A.
6	13	Solid	Mural nodule: isointense Periphery: hypointense Surrounding parenchyma: normal	Mural nodule: iso- to hyperintense Periphery: hyperintense Surrounding parenchyma: normal	No enhancement	Normal	Normal
7	136	Cystic	Mural nodule: isointense Cystic lesion: hypointense Surrounding parenchyma: normal	Mural nodule: isointense Cyst: hyperintense Surrounding parenchyma: normal	Cyst wall enhancement	Normal	N.A.

*Gd-DTPA* gadoliniumdiethylene triamine pentaacetic acid*, N.A.* not available

### Intraoperative findings

Intraoperatively, cysticercotic lesions appeared grayish-yellow or grayish-white in color with a soft texture, and the blood supply was not abundant. The lesions were well demarcated with a clear plane from the surrounding spinal cord parenchyma, and complete resection of the responsible lesion was achieved in all patients.

### Postoperative course and outcomes

All patients recovered well postoperatively. In Case 3, in addition to the responsible lesion, spinal MRI showed an intramedullary solid poorly defined lesion at the T7-T8 levels, and the patient was treated with oral albendazole (15 mg/kg) for one month. No other adjuvant therapy was performed in any patient. Considering that cysticercosis is a systemic disease, brain MRI with or without CT was requested but revealed no abnormalities in all cases. After a mean follow-up period of 56.7 ± 35.1 months, the patient’s symptoms mostly regressed.

### Representative Cases

#### Case 1

**Fig. 1:**
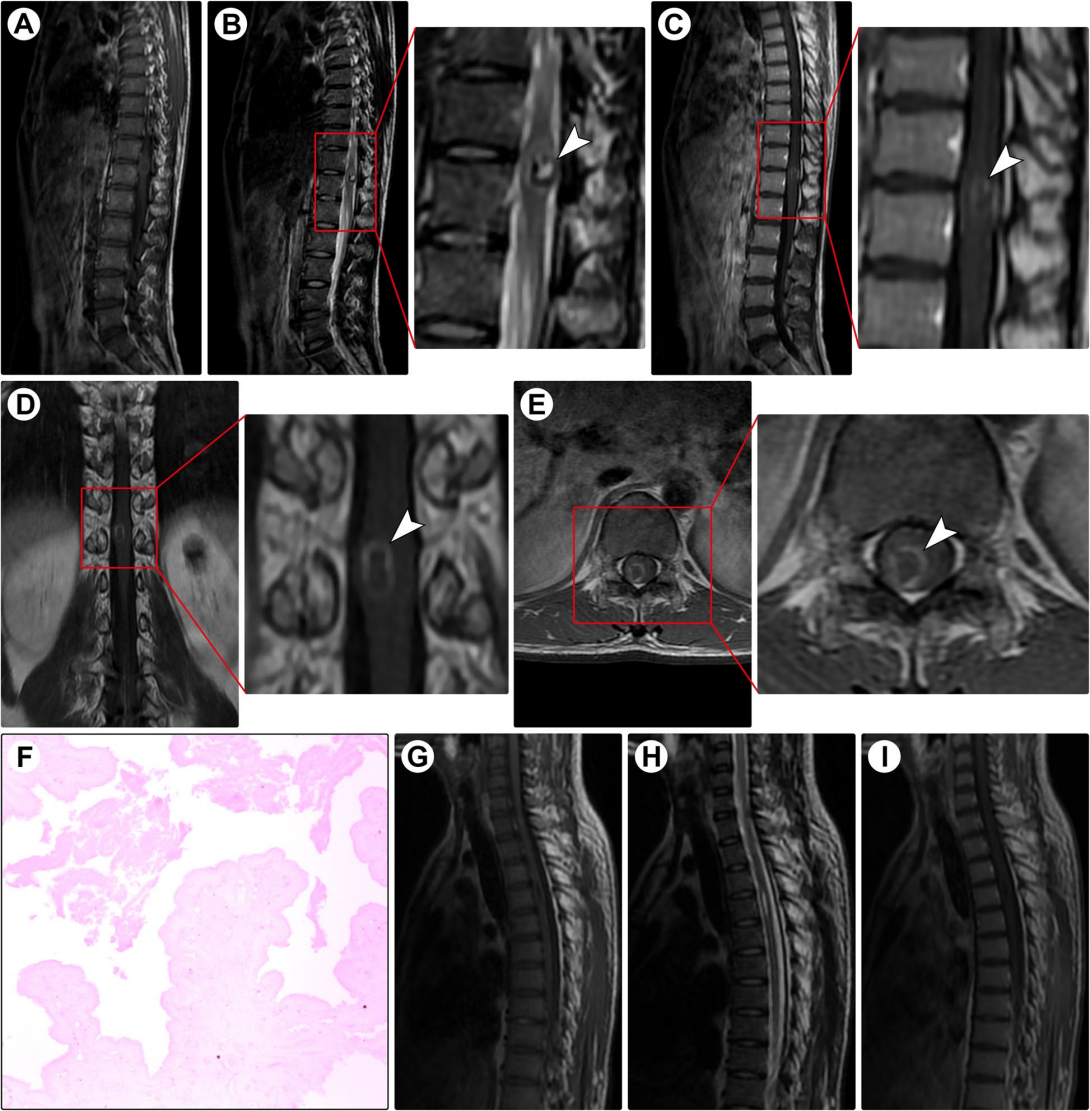
Spinal MRI and histopathology of Case 1. Spinal MRI demonstrated an intramedullary lesion (arrowheads) at the T11-T12 levels, which showed hypo- to isointensity on T1-weighted imaging (**A**) and a mushroom-like hyperintense mural nodule on T2-weighted imaging (**B**). After the administration of contrast medium, ring-like contrast enhancement was noted (**C**-**E**). Photomicrograph of the histological specimen reveals cyst wall remnants of cysticercosis (**F**; ×100). Postoperative T1-weighted (**G**), T2-weighted (**H**), and contrast-enhanced (**I**) imaging showed that the isolated lesion was completely resected

A 23-year-old man presented with a 1-year history of burning pain in the bilateral thighs and progressive numbness and weakness in both legs. Physical examination showed a loss of sensation below the T11 dermatome and muscle strength of grade 4/5 in the bilateral lower extremities. Spinal MRI revealed an intramedullary lesion at the T11-T12 levels, which showed hypo- to isointensity on T1WI (Fig. 1A) and mushroom-like hyperintensity on T2WI (Fig. 1B); after administration of contrast medium, ring-like enhancement was noted (Fig. 1C-E). A suspected diagnosis of cavernous malformation was made. Intraoperatively, a gray-yellow well-defined lesion was found and completely removed in an *en bloc* fashion. Pathological examination revealed cysticercosis (Fig. 1F). The postoperative course was uneventful, and MRI confirmed complete resection (Fig. 1G-I). During the 6 months postoperatively, the patient gradually returned to normal, and only mild sensory impairment was left after the following 7-year follow-up.

#### Case 2

**Fig. 2:**
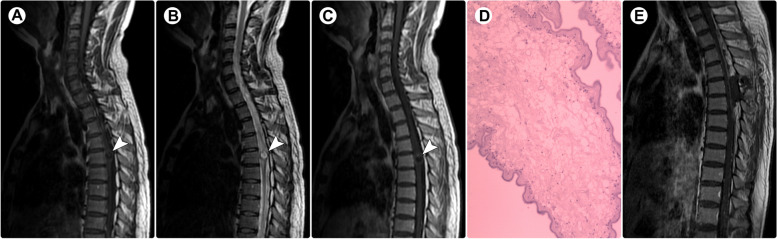
Spinal MRI and histopathology of Case 2. Spinal MRI demonstrated a cystic-solid lesion (arrowheads) within the spinal cord at the T5 level; the mural nodule was isointense on T1-weighted imaging (**A**) and T2-weighted imaging (**B**). After the administration of contrast medium, dot-like contrast enhancement was noted (**C**). Pathological examination revealed cysticercosis (**D**; ×200). Postoperative contrast-enhanced imaging confirmed complete resection (**E**)

A 24-year-old man presented with a 3-year history of back pain. Two weeks prior to admission, he developed weakness in the left leg. Physical examination showed grade 4/5 muscle strength in the left lower extremity. Spinal MRI revealed an intramedullary cystic-solid lesion at the T5 level, with a mural nodule showing isointensity on T1WI (Fig. 2A) and T2WI (Fig. 2B). After the administration of contrast medium, dot-like enhancement was noted in the mural nodule (Fig. 2C). A preliminary diagnosis of ependymoma was made. Intraoperatively, the lesion was clearly demarcated, and total removal was achieved. Pathological examination confirmed a diagnosis of cysticercosis (Fig. 2D). Brain CT and MRI showed no lesion or calcification. The symptoms were completely relieved within three months postoperatively. After a follow-up period of 7 years, the patient remained asymptomatic.

#### Case 3

**Fig. 3:**
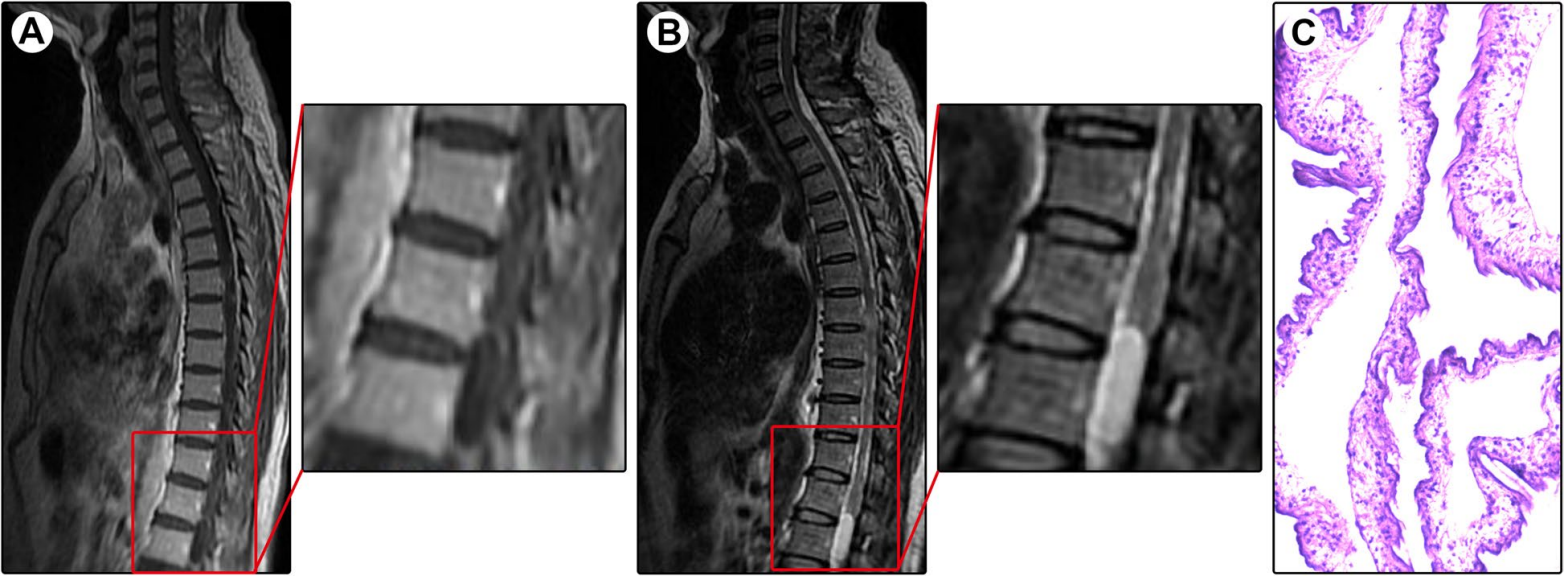
Spinal MRI and histopathology of Case 3. Spinal MRI showed an intramedullary cystic lesion (arrowheads) at the L1 level and a solid lesion (arrows) at the T7-T8 levels, both of which appeared hypointense on T1-weighted imaging (**A**) and hyperintense on T2-weighted imaging (**B**). Pathological examination revealed cysticercosis (**D**; ×200)

A 47-year-old man presented with a 2-month history of progressive numbness and weakness in both legs. One week after onset, he developed dysuria and constipation. Physical examination showed a loss of sensation below the iliac crest and grade 4/5 muscle strength in the bilateral lower extremities. Spinal MRI demonstrated an intramedullary cystic lesion at the L1 level and abnormal signals in the spinal cord at the T7-T8 levels (Fig. 3A-B). A diagnosis of enterogenous cyst with concomitant infection of the spinal cord was suspected. The lesion in the conus medullaris was surgically resected. Intraoperatively, a cystic lesion with a gray-white wall and colorless fluid was found, and it was well demarcated. Pathological examination revealed cysticercosis (Fig. 3C). Brain CT and MRI showed no abnormalities. Considering that the lesion at the T7-T9 levels may be an early-stage cysticercosis infection, oral albendazole (15 mg/kg) was prescribed. One month later, the symptoms completely resolved. The patient remained asymptomatic over a 7-year follow-up.

#### Case 4

**Fig. 4:**
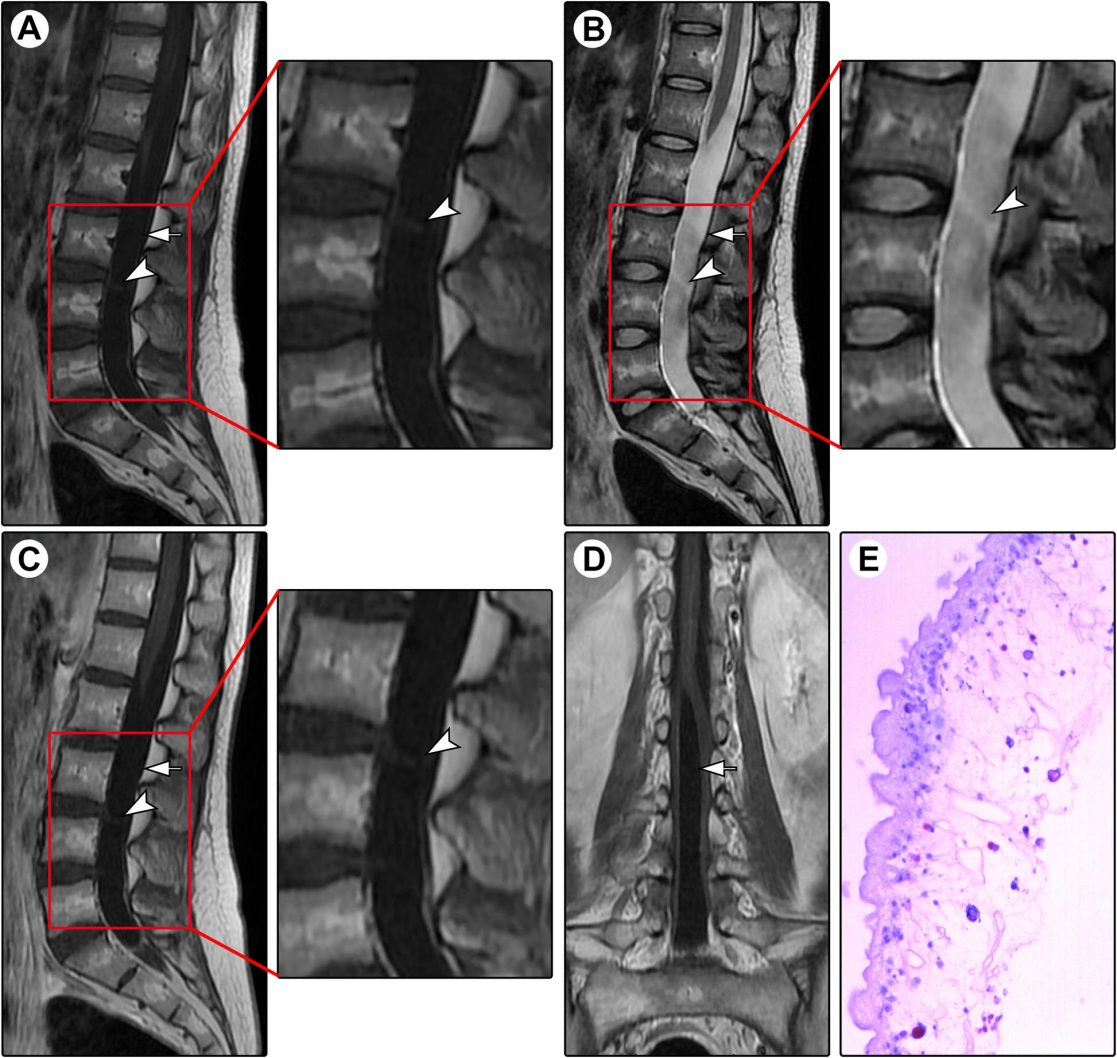
Spinal MRI and histopathology of Case 4. Spinal MRI showed a cystic lesion (arrows) at the L1-S1 levels, with a septated cyst (arrowheads) appearing isointense on T1-weighted imaging (**A**) and T2-weighted imaging (**B**). After administration of contrast medium, no enhancement was noted on sagittal (**C**) and coronal (**D**) contrasted T1-weighted imaging. Pathological examination revealed cysticercosis (**E**; ×200)

A 27-year-old woman presented with progressive back pain for half a year. The pain was aggravated after walking. The local practitioner prescribed nonsteroidal anti-inflammatory drugs, but no remarkable benefits were achieved. Five months after onset, she developed weakness of both legs and walking difficulty. After referral to our department, physical examination showed grade 4/5 muscle strength in the lower extremities. Spinal MRI showed a cystic lesion at the L1-S1 levels, with a septated cyst appearing isointense on T1WI (Fig. 4A) and T2WI (Fig. 4B). After the administration of contrast medium, no enhancement was noted (Fig. 4C&D). A diagnosis of the arachnoid cyst was suspected. Intraoperatively, the cystic fluid was light yellow. The lesion was completely removed, and the spinal cord and cauda equina nerves remained intact. Pathological examination revealed cysticercosis (Fig. 4E). Brain CT and MRI showed no lesion or calcification. Postoperatively, the patient recovered rapidly. After a follow-up period of 6 years, the patient remained asymptomatic.

#### Case 7

**Fig. 5:**
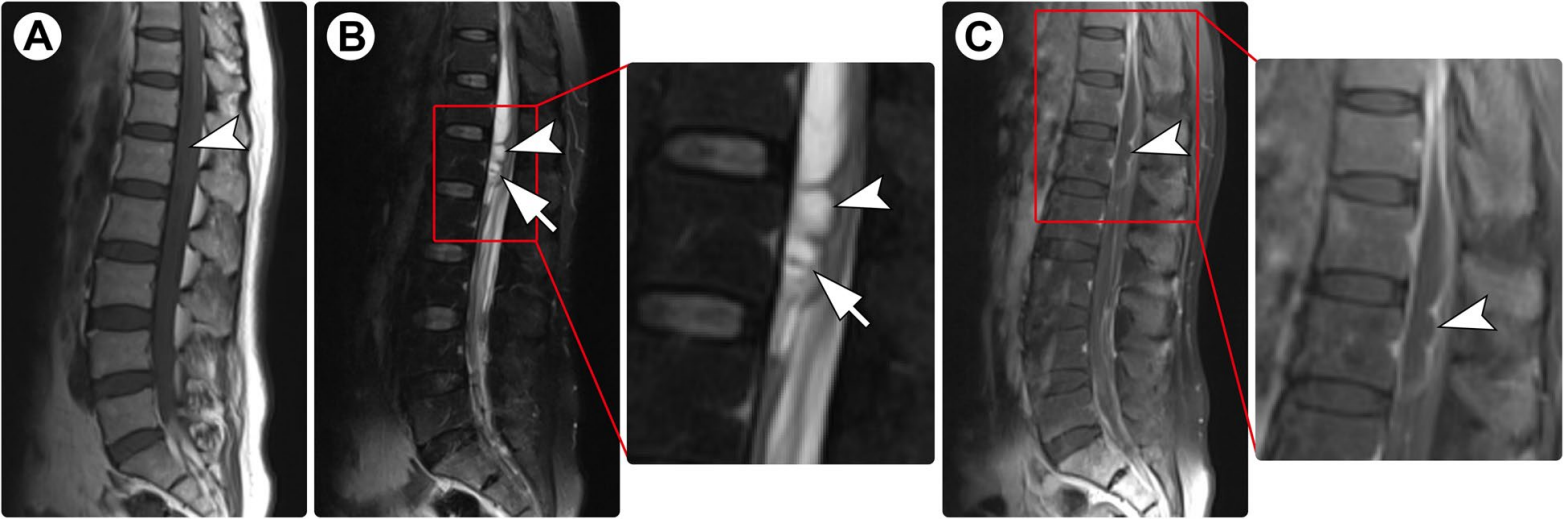
Spinal MRI of Case 7. Spinal MRI showed a septated cyst (arrowheads) at the T11-T1 levels, appearing slightly hypointensity on T1-weighted imaging (**A**) and hyperintensity on T2-weighted imaging (**B**). Additionally, an isointense mural nodule (arrows) was noted on T2-weighted imaging (**B**). After administration of contrast medium, cyst wall enhancement was demonstrated (**C**)

A 50-year-old man presented with a 3-year history of pain in the back and lower extremities. Spinal MRI showed an extramedullary cystic lesion at the T11-L1 levels. The cyst was septated, and there was a mural nodule; the cyst wall was remarkably enhanced (Fig. 5). A diagnosis of cysticercosis was suspected. Intraoperatively, a gray-yellow lesion was found, and it was well demarcated. Total removal was achieved. Pathological examination confirmed cysticercosis. The symptoms were completely relieved postoperatively, and the patient remained asymptomatic over the 10-month follow-up.

The clinical data are presented in Table 1, and the radiological characteristics of spinal cysticercosis are summarized in Table 2.

## Discussion

### Etiology and epidemiology

*Taenia solium* eggs excreted by the adult tapeworm are passed in the stool of the host, potentially contaminating the tapeworm carrier, the environment and food and water. After ingestion by humans or pigs, these eggs develop into oncospheres that can penetrate the intestinal wall and spread to other tissues. Then, the oncospheres mature into the larval stage and encyst into cysticerci. Once humans ingest pork contaminated with cysticerci, larvae are released and develop into the adult form of the tapeworm. Cysticercosis is transmitted by the fecal-oral route, including person-to-person contact, autoinfection, and contaminated food [11].

The prevalence of neurocysticercosis and spinal involvement may be underestimated since the clinical manifestations are commonly occult, and the patients may develop symptoms years after infection [12]. Before 2010, nearly fifty cases of spinal cysticercosis were reported [13–16]; nevertheless, in the last decade, only approximately ten case reports were published [10, 17–24]. Colli et al*.* proposed the hypothesis of a “sieve effect” at the transition level between the intracranial and intraspinal subarachnoid space to account for the lower incidence of spinal cysticercosis [25]. However, spinal cysticercosis can occur in either intramedullary or extramedullary regions. Intramedullary cysticercosis is mainly distributed in the thoracic spinal cord, with a few cases involving the cervical and lumbar cord. This distribution pattern supports the hypothesis that intramedullary cysticercosis occurs due to the spread of Taenia solium eggs through arterial blood because the blood supply to the thoracic cord is higher than that to the other parts of the spinal cord [10, 26]. However, extramedullary cysticercosis may result from either hematogenous transmission or subarachnoid dissemination, and it is presumed to most likely result from larval migration with cerebrospinal fluid through the ventricular system into the spinal subarachnoid space [27]. As reported, more than 50% of patients with spinal cysticercosis have evidence of *Taenia solium* infection elsewhere [28]. Additionally, approximately 75% of all spinal cysticercosis occurs in patients with an established diagnosis of intracranial cysticercosis [19, 28]. Callacondo et al. found that the frequency of spinal involvement in patients with basal subarachnoid neurocysticercosis (61%) was significantly higher than that of patients with intraparenchymal brain cysticercosis (4%). In our study, no cerebral homologous lesions or calcifications were noted.

### Diagnosis

The diagnosis of neurocysticercosis is extremely challenging. Clinical symptoms are nonspecific and location-dependent, and radiological examinations are usually not pathognomonic. Serologic tests can facilitate the diagnosis when a scolex is not visualized on neuroimaging, but the diagnostic value remains limited. Enzyme-linked immunoelectrotransfer blot (EITB) assay, based on the detection of antibodies specific for *Taenia solium* antigens, has been reported to be the best diagnostic tool with a specificity of 100% and a sensitivity of 94% ~ 98% for patients with two or more cystic or enhancing lesions; nevertheless, this assay has a decreased sensitivity (<50%) in patients with a single cysticercosis lesion [7, 11]. Additionally, serum immunoblot and cerebrospinal fluid enzyme-linked immunosorbent assay (ELISA) have also been used for the detection of cysticercal antigens or anticysticercal antibodies, while these assays are less sensitive than EITB [7]. Moreover, the presence of eosinophils in cerebrospinal fluid, which is uncommon in most infections, is also suggestive of spinal cysticercosis [29].

For solitary spinal intramedullary cysticercosis, the preoperative diagnosis is largely dependent on radiological profiles. MRI has significantly increased the detection of spinal cysticercosis in which spinal intramedullary cysticercosis manifests as a nodular or cystic lesion. Within the lesion, the scolex appears as a mural nodule with isointensity on T1WI and iso- to hyperintensity on T2WI. After the administration of contrast medium, the cysticercotic lesions show no enhancement or dot-like enhancement in the scolex, while peripheral ring-like enhancement can be noted in some cases. We considered that the perifocal abnormal signals and ring-like enhancement were attributed to reactive gliosis. In 2013, Brutto et al*.* performed a literature review that included 43 reported cases of intramedullary cysticercosis, and they found that all lesions appeared as fluid-filled cysts with hypointensity on T1WI and hyperintensity on T2WI, and most of the lesions were surrounded by parenchymal edema [30]. In 2020, Barrie and colleagues performed a meta-analysis of adult spinal cysticercosis, in which 46 articles involving 104 patients were included. A total of 24 lesions were found to be intramedullary, and 51 patients were diagnosed with primary spinal cysticercosis. However, in this systematic review, the authors did not summarize the radiological features of spinal cysticercosis [31]. Our study expanded the spectrum of MRI manifestations, and more subtle characteristics should be highlighted. The differential diagnosis of spinal intramedullary cysticercosis includes spinal cord tumors (such as astrocytoma and ependymoma), inflammatory granuloma, cavernous malformation, and cystic entities (such as arachnoid cyst and neurenteric cyst). The identification of the scolex on MRI can facilitate discrimination. In our study, due to a lack of experience, six patients were misdiagnosed with intramedullary neoplastic/angiomatous/cystic diseases, and the eventual diagnosis was based on histopathology. When we retrospectively reviewed the MRI profiles, we found that the characteristic features of intramedullary cysticercosis have great diagnostic significance.

### Treatment

Both medical therapy and surgical treatment have been reported for the management of neurocysticercosis. The recommended regimens for pharmacotherapy are albendazole (15 mg/kg/day) and praziquantel (50 mg/kg/day) for 8 days to 2 weeks, and albendazole may be superior to praziquantel in usual dosing [8]. Additionally, some authors proposed that corticosteroids should be added to the anthelmintic regimen, as perifocal inflammatory reactions may deteriorate neurological functions [15, 32]. However, some scholars hold that pharmacotherapy should only be used as an adjunct to surgery or postoperative anticysticercal treatment, considering that cysticercosis is a generalized disease [28]. Notably, patients with intraparenchymal cysticercosis may respond poorly to standard doses of antiparasitic drugs and require a longer treatment course [33]. According to the current consensus guidelines for the treatment of neurocysticercosis, surgical resection is recommended as the primary treatment for spinal intra- or extramedullary cysticercosis (evidence III) [12]. There has been no consensus on the treatment of intramedullary cysticercosis. For asymptomatic patients with spinal cysticercosis, surgical removal may be unnecessary, and high-dose steroids followed by anthelmintics can be attempted. For patients with progressive myelopathy, prompt surgical resection of the intramedullary cysticercosis should be considered to alleviate spinal cord compression. Surgical excision not only provides a definitive pathological diagnosis but also prevents irreversible spinal cord damage. With the development of microneurosurgical techniques and neuroelectrophysiological monitoring, surgical resection of a localized, well-defined intramedullary lesion can be safe. In the current study, surgical resection was performed in all patients, and clinical outcomes were favorable. The clinical prognosis of neurocysticercosis varies considerably in the existing literature. Older studies demonstrated that the prognosis was poor in cases of cysticercotic arachnoiditis treated with only symptomatic therapy, as most patients succumbed to the disease within 10 years [34]. However, intraparenchymal cerebral cysticercosis was associated with a benign prognosis [35]. Although the outcomes of spinal cysticercosis have not been clearly outlined due to the scarcity of clinical evidence, our experience indicates that patients can gain a favorable prognosis following prompt surgery with pharmaceutical anticysticercal treatment.

## Conclusions

Spinal cysticercosis is a rare cause of myelopathy. In most cases, the scolex can be seen as a mural nodule on MRI, and these characteristic imaging features can facilitate the preoperative diagnosis. Differential diagnosis of cysticercosis should be entertained in patients with spinal space-occupying lesions.

## Data Availability

Data are available from the corresponding author on reasonable request.

## Abbreviations

MRI: Magnetic resonance imaging
T1WI: T1-weighted imaging
T2WI: T2-weighted imaging
EITB: Enzyme-linked immunoelectrotransfer blot
ELISA: Enzyme-linked immunosorbent assay
